# Influence of the WHO framework convention on tobacco control on tobacco legislation and policies in sub-Saharan Africa

**DOI:** 10.1186/s12889-018-5827-5

**Published:** 2018-08-15

**Authors:** Jennifer P. Wisdom, Pamela Juma, Beatrice Mwagomba, Catherine Ndinda, Clarisse Mapa-Tassou, Felix Assah, Misheck Nkhata, Shukri F. Mohamed, Oladepo Oladimeji, Opeyemi Oladunni, Mojisola Oluwasanu, Saliyou Sanni, Jean-Claude Mbanya, Catherine Kyobutungi

**Affiliations:** 1 Wisdom Consulting, New York, NY USA; 2 African Population Health Research Centre, Nairobi, Kenya; 3 Lighthouse Trust, Lilongwe, Malawi; 4 School of Public Health and Family Medicine, College of Medicine, University of Malawi, Blantyre, Malawi; 5 Global Health Implementation Programme, School of Medicine, University of St. Andrews, St. Andrews, Scotland; 6 Human Science Research Council, Pretoria, South Africa; 7 University of KwaZulu-Natal, Durban, South Africa; 8 Department of Public Health, Faculty of Medicine and Biomedical Sciences, University of Yaoundé I, Yaoundé, Cameroon; 9 Health of Population in Transition Research Group (HoPiT), Yaoundé, Cameroon; 10 Anthropology Department, Catholic University of Malawi, Lilongwe, Malawi; 11 Department of Anthropology, Durham University, Durham, England; 12 African Regional Health Education Centre, Department of Health Promotion and Education, Faculty of Public Health, University of Ibadan, Ibadan, Nigeria; 13 School of Health Systems and Public Health, Faculty of Heath Sciences, University of Pretoria, Pretoria, South Africa

**Keywords:** Tobacco, WHO, Policy, Sub-Saharan Africa

## Abstract

**Background:**

The World Health Organization’s Framework Convention on Tobacco Control, enforced in 2005, was a watershed international treaty that stipulated requirements for signatories to govern the production, sale, distribution, advertisement, and taxation of tobacco to reduce its impact on health. This paper describes the timelines, context, key actors, and strategies in the development and implementation of the treaty and describes how six sub-Saharan countries responded to its call for action on tobacco control.

**Methods:**

A multi-country policy review using case study design was conducted in Cameroon, Kenya, Nigeria, Malawi, South Africa, and Togo. All documents related to the WHO Framework Convention on Tobacco Control and individual country implementation of tobacco policies were reviewed, and key informant interviews related to the countries’ development and implementation of tobacco policies were conducted.

**Results:**

Multiple stakeholders, including academics and activists, led a concerted effort for more than 10 years to push the WHO treaty forward despite counter-marketing from the tobacco industry. Once the treaty was enacted, Cameroon, Kenya, Nigeria, Malawi, South Africa, and Togo responded in unique ways to implement tobacco policies, with differences associated with the country’s socio-economic context, priorities of country leaders, industry presence, and choice of strategies. All the study countries except Malawi have acceded to and ratified the WHO tobacco treaty and implemented tobacco control policy.

**Conclusions:**

The WHO Framework Convention on Tobacco Control provided an unprecedented opportunity for global action against the public health effects of tobacco including non-communicable diseases. Reviewing how six sub-Saharan countries responded to the treaty to mobilize resources and implement tobacco control policies has provided insight for how to utilise international regulations and commitments to accelerate policy impact on the prevention of non-communicable diseases.

## Background

The World Health Organization’s Framework Convention on Tobacco Control (FCTC), enforced on February 27, 2005 was the first global public health treaty [1]. This treaty emerged after years of effort to spearhead an international approach to tobacco regulation that would slow the rapid growth of tobacco use. The treaty stipulated requirements for signatories to govern the production, sale, distribution, advertisement, and taxation of tobacco to reduce its impact on public health. Although the FCTC has been popular, with 180 countries currently ratifying the treaty [2], little is known about how low- and middle-income countries responded to the FCTC to modify their tobacco policies and what other contextual issues influenced the timeliness of countries’ responses [3].

The FCTC solidified tobacco use as a public health epidemic [1, 3, 4]. As evidence continues to accumulate about the global impact of tobacco consumption on non-communicable diseases (NCDs), efforts to include tobacco control have increased. The FCTC requires all participating countries to reduce this impact through various initiatives, including national programs on tobacco control; measures to protect people from second-hand tobacco smoke in public places; health warnings on tobacco products; restrictions on tobacco advertising; and prohibition of sale of tobacco products to minors [5]. Tobacco companies joined forces to oppose the FCTC and countries’ implementation of tobacco control policies, including proposing alternative language, actively lobbying against the framework, employing deception, and selectively marketing and promoting products to maintain the social acceptability of tobacco use [3, 6–8]. Some of the most devastating impacts of tobacco prevalence have been witnessed in sub-Saharan Africa, where developing nations are still struggling to fund a response to HIV and AIDS and other infectious diseases.

The history of the tobacco crop in sub-Saharan Africa today will illuminate its complex role. In the early twentieth century, a rise of African fire-cured tobacco production in the central region increased the number of Africans participating in share-cropping contracts with Europe [9, 10]. These agreements yielded financial gains for the farmers that some African countries still rely on today. Currently, Malawi is one of only two countries in the world that depend on tobacco leaf production for most of its export earnings [10]. Tobacco industries have used this reliance to their advantage in responding to a growing number of regulations and control initiatives. For example, in response to early WHO tobacco control programs at the beginning of the twenty-first century, the International Tobacco Growers Association lamented that poor African farmers [9] would suffer if tobacco regulation was successful [6]. An additional element of tobacco use in sub-Saharan Africa is its rapid growth that challenges governments to keep up with regulations to control and tax its use in the interest of protecting the public. Between 1995 and 2000, cigarette consumption increased by 38% in Africa [11]. By 2030, it is projected that 70% of the estimated 10 million global deaths from tobacco will occur in developing countries such as those in sub-Saharan Africa [11].

The WHO’s “best buys” delineate specific, low-cost, population-level interventions that, if scaled up, could reduce harmful tobacco and alcohol use, as well as unhealthy diets and physical inactivity [12]. These “best buys” hold specific promise for low and middle income countries such as many of those in sub-Saharan Africa. WHO “best buy” interventions for tobacco are, briefly, the creation of policies for tax increases on tobacco products, smoke-free indoor workplaces and public places, bans on tobacco advertising, promotion and sponsorship, and health information and warnings. Significant examinations of global tobacco policy (e.g., the International Tobacco Control Policy Project [13], have generally not included African countries, and given recent findings that policy implementation of the FCTC demand reduction measures is associated with reduction in tobacco use [14], it is especially important to assess the adoption of tobacco policies in Africa [11].

This paper describes how six sub-Saharan countries responded to the FCTC call for action on tobacco control by detailing the context, timelines, key actors, and strategies in the formulation and implementation of policies in response to the FCTC. Understanding these elements can provide insight for accelerating international mobilization to reduce and prevent NCD prevalence. In each country we present the (a) tobacco situation (production and use), (b) the FCTC adoption process (year of ratification and period taken to come up with a policy, what influenced the process), (c) the actors and industry involvement, (d) implementation of FCTC policies and WHO best buys for tobacco control.

## Methods

### Methods overview

The Analysis of Non-Communicable Disease Prevention Policies in Africa (ANPPA) study [15] employed a multiple-case study design [16] to assess policy and practice for all WHO “best buy” interventions on tobacco use, unhealthy diet, physical inactivity, and harmful alcohol use in six sub-Saharan countries: Kenya, Malawi, Nigeria, Cameroon, South Africa, and Togo. The ANPPA study was designed to generate evidence on how—and the extent to which—multi-sectoral action informs policy formulation and implementation of NCD prevention “best buy” interventions. Walt and Gilson framework of policy analysis [17] was used to guide ANPPA. The framework acknowledges the non-linearity of the policy process as well as the incremental nature of policy-making. Walt and Gilson’s framework focuses on four factors: policy (a) content, (b) actors, (c) processes, and (d) context [17].

This paper reports only on data collected on tobacco. Each country, therefore, becomes a case with its own unique approach to ratifying the FCTC and establishing policies to support tobacco control. For each case, we apply the same methods to identify differences in each country’s processes. Each case includes two primary sources of data: (1) a review of documents related to the policy formulation process and (2) key informant interviews with informants who either participated or should have participated in the policy process.

The ANPPA study was coordinated by the African Population and Health Research Center (APHRC). See Juma et al. [15] for more information on the application process and study teams.

### Document reviews

Teams conducted document reviews to describe the policy context and content, identify existing policies for their consistency with WHO “best buy” interventions, and understand the policy development processes and implementation status. Policy documents included were those that focused on NCD prevention (including acts and laws, strategic plans, guidelines, and government directives), reviews and case studies of multi-sectoral action (MSA) in successful policy formulation and implementation at a national level. Examples of policy documents included are: ministry website materials such as policy documents, strategic plans, program plans, guidelines, protocols; parliamentary records, or debates; local print media for references to policy changes, often as part of speeches by government officials; meeting minutes, activity reports, and drafts of policy statements, internal and external memos, meeting agendas, and other communications; academic journal articles; and relevant donor or non-governmental organization and development partner websites for NCD program reports. Researchers extracted data from documents including the years in which relevant policy changes occurred and the events leading up to those decisions. Some key documents date back to the 1970s (e.g., national plans and reports).

### Key informant interviews

Key informant interview participants were selected based on their expected or actual role in each country’s NCD policy formulation and implementation. Participants were selected using a combination of purposive and snowball sampling [18]. First, a broad segment of sectors (e.g., health, education, finance) and institutions (e.g., ministry officials, directors) were identified for inclusion. Next, appropriate individuals within those sectors and institutions were identified to purposively include both government and non-government (e.g., community organization, industry) actors. After key informants were identified, researchers asked them to identify additional prospective study participants who had knowledge of policy formulation and implementation. Participants were contacted through an initial telephone or email contact, and a total of 202 were interviewed across six countries [19].

APHRC and the study teams collaboratively developed interview guides during the first methodology workshop. Interview guides were informed by Walt and Gilson’s framework of policy analysis [17] and included questions for each of the four key “best buy” interventions, including the context in which the policy was developed, the policy content, actors involved in the process, and the implementation status of each policy. In addition, questions addressed how MSA was employed (or not), the processes undertaken to ensure MSA, the challenges encountered, what worked, and what did not work. During field-worker training, each team piloted the guide to obtain feedback on the questions and interview structure, and the interview guide was revised based on feedback from the pilots. Each country then used the final interview guide with minor adjustments to fit their context if necessary.

Prior to the interview, the interviewer explained the purpose of the study, risks and benefits to participating, the right to withdraw at any time without penalty, and confidentiality; participants provided verbal or written documentation of consent to participate and to be digitally recorded.

Transcripts were uploaded into the qualitative data management software NVivo coded using a codebook based on Walt and Gilson’s framework of policy analysis [17] and the framework method of qualitative analysis, a type of thematic or content analysis which guides the application of a framework to analyse qualitative data [20]. Data associated with tobacco policy formulation from all six countries were analysed for this multiple case study.

## Results

The results section summarizes each country’s activities and policies, factors shaping policy development and implementation, key actors, and quality of policies with regard key informants’ perceptions about the role of the FCTC in each country. WHO “best buy” interventions are listed in Table 1, with countries’ timelines for FCTC ratification and policy adoption in Table 2.

**Table 1:** WHO-recommended tobacco “best buy” interventions and country policy status

“Best buy” interventions	Interventions implemented	Country					
		Cameroon	Kenya	Malawi	Nigeria	South Africa	Togo
Taxation	Taxation on all cigarettes	Yes	Yes	Partial	No	Yes	Yes
	Any increase in tobacco taxes since 2011 OR in 2011 you were already at 75%	Yes	Yes	No	No	Yes	Yes
	The tax on tobacco is at least 75% (from FCTC)	No	Yes	No	No	Yes	No
	The tax applies to all tobacco products (cigarettes, snuffs, chewing tobacco) (some products = partial)	Partial	Yes	Partial	No	Yes	Yes
Smoke free policies	There is a national smoke free policy that covers all public places (some cities or settings = partial)	Partial	Partial	No	Yes	Yes	Yes
	There are enforced penalties for non-compliance (having penalties but not enforced = partial)	No	No	No	Partial	Partial	Partial
Health warnings on tobacco products	Multiple warnings/images are rotated from time to time, applies to all brands/products	No	Partial	No	Partial	Partial	No
	Large, clear, visible (at least 30% coverage) and legible all brands/all prodcuts (if only some of these words are in the legislation = partial)	Yes	Yes	No	Yes	Yes	Yes
	Health warning includes pictures or pictograms all brands/all products	No	Yes	No	No	Yes	No
	Include constituents and emissions of tobacco (e.g., how much tar) on all brands/products	Partial	Yes	No	No	No	Yes
	In official country language on all brands (only some brands/products = partial)	Partial	Partial	No	No	Partial	Yes
	Required on all tobacco products (if on only some products or brands, partial)	Partial	Yes	No	Partial	Yes	Yes
Advertising ban	Ban advertising, promotion and sponsorship of all tobacco products	Partial	Yes	No	Yes	Yes	Yes
	Ban for all forms of mass media	Yes	Yes	No	Yes	Yes	Yes
	Disclosure of expenditure on advertising by industry	No	No	No	No	No	Yes

**Table 2:** Implementation of tobacco legislation in relation to FCTC ratification^a^

Best buys	1988–1999^b^	2006	2007	2008	2010	2012	2013	2014	2015	At least one policy as of 2015
Protect people from tobacco smoke	C,S,N	N	C,K, S,N	S,N	K, T,N	C, N	T,N	C, N	C, N	C,K,N,T,S
Enforce bans on tobacco advertising	S,N	C,N	K,S,N	S,N	T,N	N	N, T	N	N	C,K,N,T,S
Warn about the dangers of tobacco	S,S,N	N	C,K, S,N	S,N	K, T,N	N, T	T,N	N	N	C,K,N,T,S
Increase taxes on tobacco	S,S	N	K, N	N	T,N	K, N, T	T,N	C, N	N	C,K,N,T,S
Ban school sales	S,C,N									C,S,N
Ban school use	C,N		C							C,N
Prevention activities	N		C							C,N

Country w/ ratification/acceptance year

^a^*C* Cameroon (3 Feb 2006), *K* Kenya (25 June 2004), *M* Malawi (not ratified), *N* Nigeria (20 Oct 2005), *T* Togo (15 Nov 2005), *S* South Africa (19 Apr 2005)

^b^No policies were implemented in 2000–2005, 2009, or 2011

### Cameroon

#### Timeline and resulting policy

Cameroon’s first tobacco control policy was formulated in 1988 [21]. After the World Health Assembly in May 2003 to adopt the FCTC, but prior to its formal ratification, the government decided to spearhead a national tobacco control program. In 2004, the Ministry of Public Health created a multi-sectoral expert platform for multiple exchanges on tobacco control called “GROUPE” by ministerial decision No. 00615/D/MSP/DPS of 11 February 2004 [22]. This group included experts from different ministerial and administrative departments [23]. In February 2006, Cameroon ratified the FCTC leading to the convening of the first national multi-sectoral meeting on tobacco under the Prime Minister’s auspices with the goal to identify and develop policies for tobacco use. Although this meeting led to individual sectors implementing smoke-free zones, Cameroon did not develop a comprehensive national policy for smoke-free zones. The “GROUPE” later developed a national comprehensive law project on tobacco, using FCTC recommendations, which was revised in 2012 and submitted to the Presidency of the Republic for issuance to the Parliament. It remains under consideration at the time of this writing.

#### Factors shaping policy ratification

Cameroonian government still grants large subsidies to Cameroon’s tobacco farmers, and tobacco is a primary crop. Although the formulation of tobacco control policies was driven by epidemiological data on tobacco use, with an emphasis on the problem of smoking and delinquency among youths and in school settings, a strong health sector was met with low visibility by civil society organizations and a lack of political will by other government sectors to implement a unified national policy.

#### Key players

The major actors involved in formulating and implementing tobacco use prevention policies were ministerial departments of Health, Trade, Education, Communication, and Finance. Although one NGO, LUTOMA (Association for the Fight against Drug Addiction and Mental Illness) provided data on the adverse effects of smoking/delinquency among youths in secondary school settings, we observed a low visibility of civil society organizations, NGOs and academics. Industry was generally not engaged in formulating tobacco control policies, but rather in the raising of tobacco taxes. Where necessary, the implementation process was discussed with industry, specifically for the policy on health information and warning on tobacco packages.

#### Quality of the policy

Cameroon’s process led to the formulation of 12 tobacco control policies which incorporated all the tobacco use prevention “best buy” interventions: tax increases on tobacco products, smoke-free indoor workplaces and public places, bans on tobacco advertising, promotion and sponsorship, and health information and warnings. Cameroon’s response to the FCTC call for action on tobacco control has been positive and addresses some “best buys,” but is still insufficient because a more comprehensive and integrated tobacco control law remains to be enacted. The tobacco control project law has been pending at the Presidency of the Republic since 2012, and study participants reported that this has been a strong hindrance to fully implementing tobacco control policies in the country. Furthermore, participants indicated the government’s tobacco farmer subsidies, which are passed through Parliament, are likely contributing to the delay in passing the comprehensive law [24]. Cameroon has at least one policy for each of the FCTC best buys: protect people from tobacco smoke, enforce bans on tobacco advertising, warn about the dangers of tobacco, increase taxes on tobacco, ban school sales, ban school use, and prevention activities (See Table 2).

### Kenya

#### Timeline and resulting policy

In Kenya, tobacco control efforts started in 1992 when Kenya first participated in the World Tobacco Day campaigns. The first tobacco control bill was drafted in 1998, well before the FCTC, but since it was a pre-FCTC bill, it did not fully address the WHO “best buy” interventions. In 2003, the focus shifted to ratification of the FCTC. In 2004, Kenya made history by being the second country (after Norway) to ratify and sign the WHO FCTC on the same day. The signature and ratification played a major role in adopting the Tobacco Control Act applying the FCTC recommendations to Kenya, which was passed in 2007 [25].

#### Factors shaping policy ratification

Tobacco control policy formulation in Kenya was largely driven by the WHO’s promotion of the FCTC, which catalysed the process in Kenya. However, strong local evidence on the economic cost of tobacco provided the needed impetus for actors in different sectors to advocate for the policy. Similarly, the availability of evidence in the public domain on the harmful effects of tobacco growing and production on the environment also contributed to the holistic argument for control. Noteworthy is the strong political influence that provided the needed push for the signing and ratifying the FCTC bill [25]. Therefore, a combination of strong global, local, economic, and political factors contributed to Kenya’s formulation of the tobacco control policy.

#### Key actors

Key actors in the Kenyan tobacco policy process were the Ministry of Health, Tobacco Control Board (which consists of various sectors relevant to tobacco control including the Ministry of Health) and civil society organizations. The policy process was a consultative approach from multiple government sectors and other stakeholders, including the private sector. Tobacco industry interference has played a key role in delaying the formulation of some key elements and poor implementation.

#### Policy quality

Together, Kenya’s policies on tobacco control are comprehensive and address all of the FCTC “best buy” interventions of tax increases; smoke-free public spaces; ban of tobacco advertising, promotion, and sponsorship; and health information and warnings. Implementation is still in the early stages. Moving forward, continuous engagement with all relevant stakeholders and allocating adequate resources (both human and financial) to support the process will be critical in the implementation stages. Kenya has at least one policy for most of the FCTC best buys: protecting people from tobacco smoke, enforcing bans on tobacco advertising, warning about the dangers of tobacco, and increasing taxes on tobacco, but does not have policies for banning school sales, banning school use, or prevention activities (See Table 2).

### Nigeria

#### Timeline and resulting policy

The development of tobacco control policies and legislation in Nigeria dates to its 1951 revenue allocation document on licensing and controlling tobacco importation [26]. The first significant effort at controlling tobacco use for public health benefits was the Tobacco Smoking (Control) Decree 20 of 1990 by the military government; the decree included measures for protection from second-hand smoke, health warnings and labels, and enforcing bans on advertising and promotion, but not taxation of tobacco products. With the transition of the Nigerian government to democratic rule in year 2000, the document’s name was changed to the Tobacco (Control) Act 1990 in line with political conventions that prohibited governance through decrees in democratic dispensations; the policy content remained unchanged, however, and guided tobacco control in Nigeria for more than two decades. The Act was weak and poorly implemented [26, 27], and government actions on tobacco control were inconsistent. For instance, although the Act emphasizes reduction in tobacco use for public health, the government still supported increases in tobacco growth as when, in 2001, the Nigerian government signed a memorandum of understanding with British American Tobacco Nigeria to build potential for regional export and significantly increase the quantity and quality of locally grown tobacco [28].

Nigeria signed the FCTC on 20 October 2005, further driving interest in tobacco legislation. In 2009, civil society organisations and several elected officials advocated for a National Tobacco Control bill [29]; after two years of consideration, the National Tobacco Control bill was passed at the Senate and House of Assembly but the presidency failed to assent to the bill. Subsequently, another version of the bill was facilitated by the Federal Ministry of Health and passed as an Executive Bill to the Federal Executive Council and Senate for approval. It was eventually approved by Nigeria’s outgoing president, Goodluck Jonathan, as the National Tobacco Control Act 2015 after mounting pressure from tobacco control advocates and despite stiff resistance from the tobacco industry. Indeed, participants indicated the outgoing president decided to sign the bill into act a few days before his exit to appease the tobacco advocacy groups and other stakeholders and avoid a reprisal from the tobacco industry [30].

#### Factors shaping policy ratification

Although there was agreement and strong advocacy among many civil society organisations, senators, elected officials, and others regarding the need for tobacco control legislation, counter-lobbying of the tobacco industry [30–32] and objections from some government ministries/agencies that felt they should lead the tobacco control efforts created dissention that impacted policy development and ratification. Indeed, the final tobacco control act was only signed by an outgoing president as he was leaving.

#### Key players

In 2009, civil society organisations advocated for a National Tobacco Control bill that was sponsored by Senator Olorunnimbe Mamara. Professor Babatunde Osotimehin, former Minister of Health, and Senator Jibrin Aminu spoke publicly in support of the proposed legislation [29]. The bill also received strong support from international and Nigerian civil society groups such as the Environmental Rights Action/Friends of the Earth, Nigeria; the Nigerian Tobacco Control Alliance; and the Coalition Against Tobacco.

#### Quality of the policy

The National Tobacco Control Act 2015 is a comprehensive, FCTC-compliant legal instrument that addresses all tobacco “best buy” interventions as well as other measures relating to reducing tobacco demand and supply as well as related matters on tobacco product specifications [33]. Several tobacco related policy documents developed after FCTC ratification are the Nigerian National Policy and Strategic Plan of Action on NCDs, developed by the Federal Ministry of Health in 2013 and reviewed in 2015, and the 2014 Standard for Tobacco and Tobacco Products–Specifications for Cigarettes, which was developed by the Standards Organization of Nigeria. These documents align with the stakeholder involvement provisions and measures outlined in the FCTC. One exception is the 2014 Standard for Tobacco and Tobacco Products—Specifications for Cigarettes, which was developed with the involvement of the tobacco industry involvement, contrary to FCTC recommendations. Nigeria has at least one policy for each of the FCTC best buys: protect people from tobacco smoke, enforce bans on tobacco advertising, warn about the dangers of tobacco, increase taxes on tobacco, ban school sales, ban school use, and prevention activities (see Table 2).

### Malawi

#### Timeline and resulting policy

Five Malawian policy documents are related to tobacco or smoking: the Tobacco Act (1970) (Last Amended in 1990) Chapter 65:02 of the Laws of Malawi [34]; the National Drug Control Master Plan for Malawi (2005); the Malawi Health Sector Strategic Plan (2011–2016) [32]; National Action Plan for Prevention and Management of Non Communicable Diseases in Malawi (2012–2016) [35]; and the Tobacco Industries Bill of 2012 [36]. Apart from the overarching NCD prevention-related policy documents [37, 38], Malawi has no specific tobacco control policies that recognize tobacco as an underlying cause of NCDs or which call for tobacco control regulation as a way to improve public health. The Tobacco Act (1970) (Last Amended in 1990) and the Tobacco Industries Bill (2012) only regulate tobacco production and sales through enhancement of agricultural practices for tobacco production and through licensing of tobacco growers, transporters, sellers, and buyers [39, 40].

#### Factors shaping policy ratification

The Malawi economy is highly reliant on tobacco, which is considered a “strategic crop” [34]. There are several barriers to the ratification of the FCTC. Tobacco farmers and industry officials oppose tobacco control. Participants indicated that there is a perception by the government that ratifying parts of the FCTC to improve health (e.g., limiting exposure to tobacco smoke) will compel them to implement all aspects of the FCTC, including Articles 17 and 18, which discourage support for tobacco farming, and that this will reduce tobacco production and negatively impact the national economy. Further, Malawi’s low prevalence of smoking and high tobacco exports led some to minimize tobacco as a major public health problem warranting legislation and policies like the FCTC. Participants also cited the influence of the tobacco industry as a barrier to FCTC ratification and implementation.

#### Key players

There is some support for ratification of the FCTC by advocacy groups, including the Tobacco Tenants and Allied Workers Union of Malawi, that wrote the president in support of reduction of reliance on tobacco crops [38], and Drug Fight Malawi, an NGO that attends international meetings on the FCTC and advocates within Malawi for tobacco control. While some government actors (e.g., the NCDs program at the Ministry of Health) favor tobacco control for promoting public health, others (e.g., the Tobacco Control Commission) prioritize tobacco farmers’ livelihood and country revenue from tobacco taxes over public health. Study participants, mostly from the health sector and some tobacco industry representatives, and the Tobacco Control Commission unanimously agreed on the need to ratify the FCTC. They noted that ratification would allow Malawi’s voice to be heard and “fight [for tobacco control] from within.”

#### Quality of the policy

Malawi has not yet acceded to or ratified the FCTC [2]. Even bills drafted after the FCTC came into force as a global treaty (e.g., the Tobacco Industries Bill of 2012) have no interventions intended to limit tobacco production or use. Thus, Malawi policies are not in line with FCTC requirements and specifically do not respect FCTC Articles 17 and 18 that require countries to focus on “provision of support for economically viable alternative activities for tobacco growers” and “protection of the environment and the health of persons,” respectively [1]. Study participants confirmed that Malawi has no specific public health-related tobacco control policies. Some sectors have attempted to use other laws to lobby for implementing some of the tobacco control interventions consistent with the FCTC requirements. For example, one civil society organization attempted to compel the government to ban public smoking by citing the Environmental Management Act 1996 [37]; the courts referred it back to the government executive branch, which said it was in the process of developing a public smoking policy. However, the mentioned policy had not been seen by any of the study participants by the time of data collection. Some participants intimated that Malawi could soon ratify the FCTC and indicated this will likely be advantageous to tobacco control since the majority of the FCTC articles might be implemented without much contention; for the two contentious articles (FCTC Articles 17 and 18), Malawi could learn from experiences of other countries that have ratified the convention but are still in the transition period to reduce overreliance on tobacco crops.

### South Africa

#### Timeline and resulting policy

On *World No Tobacco Day* in 1993, Nelson Mandela called on the apartheid government under F.W. de Klerk to pass tobacco control legislation. In response, the Tobacco Control Act was passed in 1993 but due to the then-apartheid government’s strong ties with the tobacco industry, the Act did not result in major shifts in tobacco smoking. Although the Act was passed in 1993, it was under the democratic government in 1994 that the Tobacco Control regulations were drafted [41, 42]. With the introduction of democracy in 1994, the African National Congress (ANC) brought both a notable distance from tobacco industry influence and specific health champions in the form of the president Nelson Mandela and Health Minister Dr. Nkosazana Dlamini-Zuma who showed serious commitment to public health, including tobacco control. Over the next few years, the ANC called for smoke-free cabinet meetings, called out tobacco companies that failed to display health warnings clearly on cigarette packs, introduced a 50% tax on the retail price of cigarettes, and in 1999 amended the Tobacco Products Control Act that outlawed smoking in public buildings, banned tobacco advertising in all its various forms, and made it illegal to sell cigarettes without health warnings on the pack.

#### Factors shaping policy ratification

South Africa was on the forefront of African countries to implement strict tobacco control policies. The ANC’s rise to power and its recognition of tobacco as a racial equity issue were a strong counterforce to tobacco industry efforts to reduce control policies.

#### Key players

The ANC produced substantial pressure to improve tobacco control policies, as a matter of racial equity. Consistent tobacco industry opposition resulted in a weaker version of the Tobacco Products Control Act in 1993, with multiple amendments to attempt to strengthen it in 1999, 2003, 2007, and 2008 [41].

#### Quality of the policy

So far, South Africa has put in place the legislative framework and has implemented the WHO “best buy” interventions. The Tobacco Control Act and subsequent amendments prohibit smoking at indoor working places, washrooms, or any other places frequented by employees. These provisions do not prohibit smoking in private dwellings, which is in itself a fallacy, given that many women are domestic workers in private homes and remain unprotected from secondary exposure [42]. Subsequent amendments prohibited smoking in public places and vehicles with children as passengers and advertising and promotion of tobacco products. Amendments also restricted the distribution of tobacco products and advertising at the point of sale. Tobacco manufacturers were required to have clearly printed health warnings on the cigarette packets. South Africa has at least one policy for most of the FCTC best buys: protect people from tobacco smoke, enforce bans on tobacco advertising, warn about the dangers of tobacco, increase taxes on tobacco, and ban school sales, except for banning school tobacco use and implementing prevention activities (see Table 2).

### Togo

#### Timeline and resulting policy

The development of tobacco control policies and legislation dates to its 2009 public health law under Articles 89 to 93 related to the fight against social scourges, including harmful use of alcohol, tobacco use, substance abuse, and prostitution. This law, however, focused largely on regulating (but not banning) tobacco advertising (Article 90), warning about the dangers (Article 91), and banning smoking in public places (Article 92). The law did not address raising taxes on tobacco and was not highly enforced. Significant efforts at controlling tobacco use started with the ratification of the FCTC on 15 November 2005.

#### Factors shaping policy ratification

Togo is a low-income country with few tobacco retailers. Participants said that because Togo is neither a tobacco producer nor manufacturer, it was relatively easy for policy-makers to use available evidence in the public domain on tobacco’s harmful effects to achieve convergence about the nature of the problem, policy, and politics and to convince the government to act for tobacco control. Togo’s policies aim to improve the health of vulnerable groups, to reduce health gaps between the most and least vulnerable groups, and to flatten the social gradient in health across the entire population.

#### Key players

The Ministry of Health played a key role in tobacco policy implementation. Participants reported that the tobacco industry strongly opposed tax increases on tobacco products and said the industry repeatedly attempted to delay or dissuade policies’ enactment and implementation.

#### Quality of the policy

Togo passed comprehensive national legislation, but it is not fully WHO FCTC compliant because fully compliant health warnings were removed from the version of the law parliament approved, although there is some policy for health warnings. Togo has at least one policy for most of the FCTC best buys: protect people from tobacco smoke, enforce bans on tobacco advertising, warn about the dangers of tobacco, and increase taxes on tobacco, except for banning school sales, banning school tobacco use and implementing prevention activities (see Table 2).

## Discussion

Countries varied widely on their timelines for addressing tobacco control, with the earliest tobacco-related policies in the mid-twentieth century (e.g., Nigeria, 1951; Malawi, 1970) and others developing policies only around the time of the FCTC in the mid-2000s (Togo). Earlier attention to tobacco-related policies often was more about commerce and agriculture than public health and did not relate to the country’s implementation of FCTC policies (e.g., Malawi has not yet ratified the FCTC). Most countries developed piecemeal legislation starting in the 1990s and early 2000s, with the FCTC providing a strong boost as countries began to ratify it in the mid to late 2000s. The process for adopting tobacco policies focused on public health and consistency with “best buy” interventions was greatly motivated by FCTC adoption. It brought significant international focus on tobacco; countries’ treaty signing provided additional energy for the study to approve legislation so as not to be seen as falling behind on the world stage.

Countries demonstrated high variability in their socio-political contexts: (a) tobacco was a significant cash crop that contributed to national employment and revenue (Cameroon, Malawi), (b) leadership demonstrated connections or interests in tobacco industries that reduced political will for tobacco control (Nigeria, apartheid South Africa, Togo), (c) limited resources were available for NCD prevention, given countries’ political upheaval or communicable disease challenges (Cameroon, Kenya, Malawi, Nigeria, South Africa, Togo), (d) specific high-profile champions in government advocated for tobacco control (South Africa, Kenya, Nigeria), and (e) tobacco industries and their interests were strongly against tobacco control legislation (Cameroon, Kenya, Malawi, South Africa, Nigeria). This variability in contexts also influenced the countries’ success implementing FCTC and WHO “best buys” on tobacco control.

Multiple actors engaged in deliberations about tobacco policies [19]. Most countries’ health ministries led the process of evaluating FCTC guidelines and preparing policies with other ministries, NGOs, civil society organisations, and academics [23, 25, 41, 43, 44]. These groups generally supported tobacco control policies. The extent to which stakeholders were involved varied from nominal involvement to providing data, testimony, or lobbying for country-specific measures.

Countries struggled with whether or how to involve the tobacco industry in their selected stakeholder meetings; for instance, in South Africa, the constitution requires stakeholder representation, and the tobacco industry sued to require the government to recognize its inclusion as a stakeholder [41]. All countries reported significant interference from the tobacco industry in enacting tobacco control policies, with some countries also having labour groups or tobacco farmers opposing tobacco control policies (Malawi, Nigeria). This is similar to industry efforts to derail the FCTC prior to enactment and in low- and middle-income countries since FCTC enactment [7, 8].

Most countries have addressed all four WHO “best buy” interventions, although not necessarily to the extent the FCTC recommends. Countries have been delayed by internal political challenges, conflicts of interest among leadership, tobacco industry interference, and limited resources. This is similar to other reports of progress among low- and middle-income countries that have struggled with implementing “best buy” interventions, especially related to tobacco [4, 5, 29].

Overall, the WHO FCTC has been enormously useful in reducing tobacco use and its health effects. Despite significant tobacco industry opposition, it remains the only international health treaty and has demonstrated success in reducing NCDs internationally. It is highly advisable to continue to improve both science and practice on how best to implement country-level implementation of international health treaties. Recommendations include: (a) how to best implement multi-sectoral action to ensure relevant stakeholders are included and their needs considered, (b) how politicians and civil service organizations can address tobacco industry engagement and interference, (c) how to address within-country conflicts of interest such as balancing needs related to tobacco’s benefits for farmers, tax revenues, and public health;, and (d) how to expand the reach of international health organizations to implement more treaties for improving global health.

## Conclusions

The WHO Framework Convention on Tobacco Control provided an unprecedented opportunity for an international governing organization to influence tobacco control interventions that lead to non-communicable disease prevention worldwide. Reviewing how six sub-Saharan countries responded to the treaty to mobilize resources and implement tobacco control provided insight for how to accelerate international mobilization to prevent non-communicable disease.

## Abbreviations

ANC: African National Congress
ANPPA: Analysis of Non-Communicable Disease Prevention Policies in Africa
APHRC: African Population and Health Research Center
FCTC: Framework Convention on Tobacco Control
NCD: Non-communicable disease

## References

[1] World Health Organization. WHO framework convention on tobacco control. Geneva; 2003.

[2] World Health Organization (2017). Parties to the WHO Framework Convention on Tobacco Control.

[3] World Health Organization. History of the World Health Organization framework convention on tobacco control. Geneva; 2009.

[4] World Health Organization (2013). Global action plan for the prevention and control of noncommunicable diseases 2013-2020.

[5] World Health Organization Regional Office for Africa. The WHO framework convention on tobacco control: 10 years of implementation in the African region. Brazzaville; 2015.

[6] Yach D, Bettcher D (2000). Globalisation of tobacco industry influence and new global responses. Tob Control.

[7] Lee S, Ling PM, Glantz SA (2012). The vector of the tobacco epidemic: tobacco industry practices in low and middle-income countries. Cancer Causes Control.

[8] Sebrie E, Glantz SA (2006). The tobacco industry in developing countries has forestalled legislation on tobacco control. Br Med J.

[9] Green E (2012). Land concentration, institutional control, and African agency: growth and stagnation of European tobacco farming in Shire Highlands, c 1900-1940.

[10] Townsend L, Flisher AJ, Gilreath T, King G (2006). A systematic literature review of tobacco use among adults 15 years and older in sub-Saharan Africa. Drug Alcohol Depend.

[11] Nturibi EM, Kolawole AA, McCurdy SA (2009). Smoking prevalence and tobacco control measures in Kenya, Uganda, the Gambia and Liberia: a review. Int J Tuberc Lung Dis.

[12] World Health Organization and World Economic Forum (2011). From burden to “best buys”: reducing the economic impact of non-communicable diseases in low- and middle-income countries.

[13] Fong GT, Cummings KM, Borland R, Hastings G, Hyland A, Giovino GA, Hammond D, Thompson ME (2006). The conceptual framework of the International Tobacco Control (ITC) Policy Evaluation Project. Tob Control.

[14] Gravely S, Giovino GA, Craig L, Commar A, D’Espaignet ET, Schotte K, Fong GT (2017). Implementation of key demand-reduction measures of the WHO Framework Convention on Tobacco Control and change in smoking prevalence in 126 countries: an association study. Lancet Public Health.

[15] Juma P, Mohamed SF, Wisdom JP, Kyobutungi C, Oti S. Analysis of non-communicable disease prevention policies in sub-Saharan Africa: study protocol. Arch Public Health. 2016;74(25) 10.1186/s13690-016-0137-9 PMC4916543 27335640

[16] Yin RK (2013). Case study research: design and methods.

[17] Walt G, Gilson L (1994). Reforming the health sector in developing countries: the central role of policy analysis. Health Policy Plan.

[18] Boyatzis RE (1998). Transforming qualitative information: thematic analysis and code development.

[19] Juma PA, Mapa-tassou C, Mohamed S, Matanje Mwagomba BL, Ndinda C, Oluwasanu M, Mbanya J, Mkhata, Asiki G, Kyobutungi C. Multi-sectoral action in non-communicable disease prevention policy development in five African countries. BMC Public Health. 2018;18(Suppl 1). 10.1186/s12889-018-5826-6. 10.1186/s12889-018-5826-6 PMC6117629 30168391

[20] Gale NK, Heath G, Cameron E, Rashid S, Redwood S. Using the framework method for the analysis of qualitative data in multi-disciplinary health research. BMC Med Res Methodol. 2013;13(117) 10.1186/1471-2288-13-117 PMC3848812 24047204

[21] Mbede J. Decision no. 0222/D/MPH/SG/DMPHP prohibiting smoking in institutions and facilitities under the Ministry of Public Health. Ministry of Public Health; 1988. Decision.

[22] Olangena U. Decision no. 00615/MSP/DPS creating a multisectoral expert group on tobacco. Ministry of Public Health; 2004.

[23] Mapa-Tassou C, Bonono CR, Wisdom JP, Juma PA, Assah F, Ongolo-Zogo P, Fezeu L, Sobngwi E, Mbanya JC. Two decades of tobacco use prevention and control policies in Cameroon: results from the analysis of non-communicable disease prevention policies in Africa. BMC Public Health. 2018;18(Suppl 1). 10.1186/s12889-018-5828-4. 10.1186/s12889-018-5828-4 PMC6117627 30168394

[24] Cameroon Academy of Sciences. Workshop for dissemination of the NASAC expert committee on tobacco control in Africa report to stakeholders in Cameroon. Yaounde; 2014.

[25] Mohamed S, Juma PA, Asiki G, Kyobutungi C. Facilitators and barriers in the formulation and implementation of tobacco control policies in Kenya: a qualitative study. BMC Public Health. 2018;18(Suppl 1). 10.1186/s12889-018-5830-x. 10.1186/s12889-018-5830-x PMC6117633 30168390

[26] Nwhator SO (2011). Nigeria’s costly complacency and the global tobacco epidemic. J Public Health Policy.

[27] World Health Organization. WHO Report on the Global Tobacco Epidemic 2008, The MPOWER package. Geneva; 2008.

[28] British American Tobacco Company (2012). Stakeholder report 2012/2013 “shared planet, shared solutions”.

[29] Drope J (2011). Tobacco control in Africa: people, politics, and policies.

[30] Oladepo O, Oluwasanu M, Abiona O. Analysis of non-communicable diseases in Nigeria. University of Ibadan, Nigeria, Department of Health Promotion and Education.

[31] Agaku I, Akinyele A, Oluwafemi A (2012). Tobacco control in Nigeria: policy recommendations. Tob Induc Dis.

[32] Emmanuel O (2013). Nigerian anti-tobacco advocates to push for higher tobacco taxes.

[33] Federal Republic of Nigeria (2015). National Tobacco Act.

[34] Government of Malawi. Laws of Malawi, Chapter 65:02, Tobacco. 1987.

[35] Ministry of Health (2011). Malawi health sector strategic plan 2011–2016: moving towards equity and quality.

[36] Ministry of Health (2013). National action plan for prevention and management of non-communicable diseases in Malawi 2012–2016.

[37] Tobacco Association of Malawi (2014). Tobacco Association of Malawi.

[38] Tobacco Tenants and Allied Workers Union of Malawi (2014). Letter ot the President of the Government of Malawi.

[39] Government of Malawi (1996). Environment Management Act No. 23.

[40] Department of Nutrition, HIV and AIDS. Nutrition guidelines for prevention and management of dietary related non communicable diseases. Republic of Malawi; 2009.

[41] Ndinda C, Ndhlovu TP, Juma P, Asiki G, Kyobutungi C. The evolution of non-communicable diseases policies in post-apartheid South Africa. BMC Public Health. 2018;18(Suppl 1). 10.1186/s12889-018-5832-8. 10.1186/s12889-018-5832-8 PMC6117625 30168397

[42] Ndinda C, Hongoro C (2017). Analysis of NCD Prevention Policies in Africa: A Case Study of South Africa.

[43] Oladepo O, Oluwasanu M, Abiona O. Analysis of tobacco control policies in Nigeria: historical development and application of multi-sectoral action. BMC Public Health. 2018;18(Suppl 1). 10.1186/s12889-018-5831-9. 10.1186/s12889-018-5831-9 PMC6117635 30168392

[44] Sanni S, Hongoro C, Ndinda C, Wisdom JP. Assessment of the multi-sectoral approach to tobacco control policies in South Africa and Togo. BMC Public Health. 2018;18(Suppl 1). 10.1186/s12889-018-5829-3. 10.1186/s12889-018-5829-3 PMC6117630 30168399

